# Supplementary material for: Concept transfer of synaptic diversity from biological to artificial neural networks
Source: Nat Commun. 2025 Jun 2;16:5112. doi: 10.1038/s41467-025-60078-9 (PMC12130319; doi:10.1038/s41467-025-60078-9)
Supplement: Supplementary file 1 — Supplementary Information [file 41467_2025_60078_MOESM1_ESM.pdf]

Supplementary Information: Concept Transfer of Synaptic Diversity from Biological to Artificial Neural Networks

Martin Hofmann[1*], Moritz Franz Peter Becker[2], Christian Tetzlaff[2,3] and Patrick Mäder[1,4,5]

[1*]Data-intensive Systems and Visualization Group (dAI.SY), Technische Universität Ilmenau, Max-Planck-Ring 14, Ilmenau, 98693, Thuringia, Germany.
[2]Group of Computational Synaptic Physiology, Department for Neuro- and Sensory Physiology, University Medical Center Göttingen, Humboldtallee 23, Göttingen, 37073, Lower Saxony, Germany.
[3]Campus-Institut Data Science (CIDAS), University of Göttingen, Goldschmidtstraße 1, Göttingen, 37077, Lower Saxony, Germany.
[4]German Centre for Integrative Biodiversity Research (iDiv) Halle-Jena-Leipzig, Deutscher Platz 5e, Leipzig, 04103, Saxony, Germany.
[5]Faculty of Biological Sciences, Friedrich Schiller University, Fürstengraben 1, Jena, 07745, Thuringia, Germany.

*Corresponding author(s). E-mail(s): martin.hofmann@tu-ilmenau.de;
Contributing authors: kontakt@moritzbecker.com; tetzlaff@phys.uni-goettingen.de; patrick.maeder@tu-ilmenau.de;

Supplementary Information

Additional experiments and information regarding the experimentation to evaluate synaptic diversity transferred to ANNs

Predictions with Default Hyperparameters

To systematically evaluate the general impact of our proposed methods on neural networks, we conducted extensive parameter tuning experiments. The initial parameter optimization was performed on a carefully selected 1% subset of the MNIST dataset, split into a 2:1 ratio for training and validation. Using the nevergrad optimization framework [1] with a computational budget of 100 iterations, we tuned an MLP architecture over ten epochs with a batch size of 1,000. This process yielded optimal values for our key parameters: gradient scaling rate $\tau = 0.09$, rejuvenation distance factor $d_{\mathrm{re}} = 14$, and division factor $\Gamma = 2$. These parameters were then applied consistently across all subsequent experiments to ensure fair comparison.

Detailed Analysis of Training Dynamics

Figure 1 presents a comprehensive examination of how different combinations of biological modifications affect the training dynamics across three neural architectures (AlexNet, MLP, and ResNet56) and three datasets (MNIST, CIFAR10, and CIFAR100). The training curves reveal distinct patterns in learning behavior and stability across different configurations of fuzzy learning (FL), weight rejuvenation (WR), and weight splitting (WS).

In baseline configurations (FL=0, WR=0, WS=0), ResNet56 exhibits notable overfitting tendencies, particularly visible in CIFAR10 and CIFAR100 tasks where accuracy declines after reaching peak performance. This overfitting pattern persists when only weight splitting is applied. However, the introduction of fuzzy learning rates or weight rejuvenation demonstrates a clear mitigating effect on this overfitting behavior, with combinations including both FL and WR showing the most stable post-peak performance.

Architecture-specific responses to these modifications are particularly noteworthy. The MLP architecture achieves optimal performance through the combination of weight rejuvenation and weight splitting for CIFAR10 and CIFAR100, while fuzzy learning rates alone prove most effective for MNIST. AlexNet shows similar differentiation, with WR+WS yielding the best results for MNIST but preferring fuzzy learning rates for CIFAR100. ResNet56 consistently performs best with the full combination of all three modifications across all datasets.

The learning trajectories also demonstrate varying speeds of convergence. Configurations incorporating weight splitting consistently show faster initial learning phases, while the full modification set (FL=1, WR=1, WS=1) achieves the fastest overall convergence to peak accuracy. This acceleration in learning is particularly pronounced in more complex tasks, with CIFAR100 showing the most dramatic improvements over baseline performance.

These findings suggest that biological modifications not only enhance final accuracy but fundamentally alter the learning dynamics of neural networks. The synergistic effects of combining FL, WR, and WS are particularly evident in the stability of training trajectories and resistance to overfitting, especially in deeper architectures like ResNet56. This analysis complements the main results by providing a detailed view of how these biological mechanisms influence the temporal aspects of neural network training.

Our experiments on varying sampling methods for FL did not show significant differences

Tables

Tables 1, 2, 3, and 4 show the results presented in Figures 1 and 2 in the main paper.

Supplementary Table 1: Results of the experiment on accuracy. The table shows the observed mean accuracy in percent and standard deviations for 3-fold cross-validation runs for a given combination of parameters for the datasets MNIST (M10), CIFAR-10, and CIFAR-100 (C10 and C100). The highest accuracy for a dataset and architecture combination is highlighted in bold. The numbers (FL), (WR), and (WS) denote the methods of fuzzy learning rates, weight rejuvenation, and weight splitting, respectively.

Methods			MLP acc [%]↑ ± std.			AlexNet acc [%]↑ ± std.			ResNet56 acc [%]↑ ± std.		
**FL**	**WR**	**WS**	M10	C10	C100	M10	C10	C100	M10	C10	C100
			95.70 ±0.21	55.00 ±0.73	23.62 ±0.56	76.83 ±5.56	19.40 ±0.75	1.30[1] ±0.20	96.28 ±0.11	50.54 ±1.52	37.39 ±0.38
		√	96.81 ±0.23	55.73 ±0.90	**28.33** ±0.50	97.99 ±0.05	63.01 ±0.76	28.29[2] ±0.52	98.08 ±0.13	61.52 ±1.16	36.79 ±0.19
	√		96.63 ±0.19	**56.13** ±0.81	26.91 ±0.64	97.72 ±0.09	57.64 ±0.50	2.54[1] ±0.20	96.51 ±0.34	50.92 ±1.09	33.88 ±0.24
	√	√	97.18 ±0.14	54.80 ±0.96	27.12 ±0.39	98.11 ±0.06	63.01 ±0.75	**33.71**[2] ±0.83	96.38 ±0.51	50.74 ±1.68	43.13 ±1.21
√			95.72 ±0.30	55.51 ±0.72	23.43 ±0.53	76.47 ±4.18	21.03 ±0.66	0.97[1] ±0.11	**98.24** ±0.14	60.92 ±0.26	41.12 ±0.47
√		√	96.79 ±0.18	55.49 ±0.40	28.29 ±0.49	98.15 ±0.02	62.93 ±0.95	28.22[2] ±0.93	98.14 ±0.11	62.04 ±0.52	35.14 ±0.74
√	√		96.74 ±0.14	56.01 ±0.61	26.74 ±0.45	97.87 ±0.10	57.36 ±0.43	2.11[1] ±0.39	96.61 ±0.55	51.28 ±1.01	32.45 ±1.41
√	√	√	**97.25** ±0.14	54.42 ±0.60	27.07 ±0.57	**98.28** ±0.14	**63.42** ±0.89	33.14[2] ±0.16	96.20 ±0.56	**64.96** ±1.47	**44.37** ±1.26

Notes
[1] Very low stability with gradient explosion without further preventive measures
[2] High gains in stability due to weight splitting

Numerical Results on Gradient Inversion

The gradient inversion experiments were designed to evaluate the privacy-preserving properties of our biological modifications. We conducted comprehensive tests using both untrained models and networks trained for 100 epochs

Supplementary Table 2: Results of the experiment on learning speed. The table shows the observed mean epoch when the maximum accuracy occurred and its standard deviations for 3-fold cross-validation runs for a given combination of parameters for the datasets MNIST (M10), CIFAR-10, and CIFAR-100 (C10 and C100). The lowest numbers of trained epochs for dataset and architecture combinations are highlighted in bold. The numbers FL, WR, and WS denote the methods of fuzzy learning rates, weight rejuvenation, and weight splitting, respectively.

Methods			MLP ep(max acc)↓ ± std.			AlexNet ep(max acc)↓ ± std.			ResNet56 ep(max acc)↓ ± std.		
**FL**	**WR**	**WS**	M10	C10	C100	M10	C10	C100	M10	C10	C100
			99 ±0.8	99 ±0.8	99 ±1.9	99 ±0.9	93 ±5.7	93 ±1.3	99 ±0.8	98 ±1.6	99 ±0.2
		√	100 ±0.5	67 ±9.5	96 ±1.7	79 ±9.5	76 ±7.1	89 ±2.8	86 ±4.9	90 ±5.5	78 ±7.6
	√		98 ±1.7	96 ±1.7	99 ±0.9	94 ±5.3	99 ±0.9	79 ±13.0	96 ±1.6	97 ±3.1	93 ±2.0
	√	√	88 ±3.7	**46** ±3.3	**62** ±5.0	**68** ±14.3	59 ±9.0	77 ±2.9	87 ±2.1	86 ±0.5	91 ±0.5
√			**81** ±4.1	91 ±7.4	88 ±11.9	82 ±7.9	84 ±8.9	**56** ±14.3	88 ±1.4	87 ±1.6	93 ±1.7
√		√	89 ±1.41	74 ±7.78	93 ±4.97	88 ±4.11	90 ±6.94	87 ±2.36	88 ±0.47	100 ±0.47	100 ±0.0
√	√		87 ±7.3	85 ±9.5	83 ±13.6	83 ±5.4	90 ±0.5	87 ±4.2	84 ±4.5	88 ±0.8	92 ±1.7
√	√	√	86 ±1.7	55 ±7.6	**62** ±6.9	85 ±10.7	**58**[1] ±3.3	85 ±11.5	**82** ±7.9	**85**[1] ±4.5	**74**[1] ±4.2

Notes
[1] Configuration with the highest accuracy achieved with the lowest number of epochs.

on CIFAR-100, spanning multiple architectures (MLP, AlexNet, ResNet20, ResNet32) and various combinations of our proposed methods. The reconstruction error, measured as mean square error (MSE), serves as our primary metric, with higher values indicating better resistance to gradient inversion attacks. Our analysis includes baseline comparisons and examines how different combinations of Fuzzy Learning (FL), Weight Rejuvenation (WR), and Weight Splitting (WS) affect the network's resilience to reconstruction attempts. Table 5 shows the results presented in main paper Figure 5.

Prediction Performance with Tuned Hyperparameters

We extended our evaluation to state-of-the-art architectures with carefully tuned hyperparameters to assess the scalability and broader applicability of our biological modifications. The experiments encompass both image classification tasks (CIFAR10, CIFAR100, ImageWoof, Tiny ImageNet) and complex time-series prediction challenges (Lorenz'96, Thomas attractor). For image classification, we evaluated modern architectures including ResNeXt, WResNet, EfficientNet V2, SEResNeXt, and Swin Transformer V2. The time-series experiments focused on comparing traditional recurrent architectures (LSTM, GRU) with newer approaches (Transformer, FDN) across different dimensional spaces and chaotic systems. All models were evaluated both in their original form

Supplementary Table 3: Results of the experiment on accuracy. The table shows the observed normalized area under the curve (the sum of all observed accuracies during the training divided by the number of epochs) and the respective standard deviations for 3-fold cross-validation runs for a given combination of parameters for the datasets MNIST (M10), CIFAR-10, and CIFAR-100 (C10 and C100). The highest nAUC for a dataset and architecture combination is highlighted in bold. The numbers FL, WR, and WS denote the methods of fuzzy learning rates, weight rejuvenation, and weight splitting, respectively.

Methods			MLP nAUC↑ ± std.			AlexNet nAUC [%]↑ ± std.			ResNet56 nAUC [%]↑ ± std.		
**FL**	**WR**	**WS**	M10	C10	C100	M10	C10	C100	M10	C10	C100
			94.35 ±0.23	49.65 ±0.47	18.63 ±0.47	22.49 ±1.07	13.51 ±1.06	0.97[1] ±0.05	93.80 ±0.22	43.92 ±1.45	33.23 ±0.51
		√	95.76 ±0.27	**53.16** ±0.57	23.90 ±0.41	93.91 ±0.23	52.75 ±0.28	15.02[2] ±0.50	95.68 ±0.90	54.76 ±0.35	**32.50** ±0.04
	√		95.64 ±0.19	51.77 ±0.50	21.93 ±0.43	71.66 ±1.05	32.77 ±1.21	1.42[1] ±0.13	90.36 ±1.34	43.04 ±0.96	24.81 ±0.24
	√	√	96.53 ±0.13	52.77 ±0.96	**24.49** ±0.35	96.81 ±0.05	57.64 ±0.61	**24.60**[2] ±0.48	89.90 ±1.07	42.18 ±1.21	25.21 ±0.69
√			94.33 ±0.27	49.70 ±0.62	18.55 ±0.41	27.34 ±1.50	14.89 ±0.68	0.92[1] ±0.06	**95.19** ±0.29	54.58 ±0.46	25.77 ±1.13
√		√	95.75 ±0.16	53.15 ±0.45	23.82 ±0.42	93.82 ±0.39	52.41 ±0.75	14.54[2] ±0.42	94.82 ±0.66	53.83 ±0.77	21.22 ±1.22
√	√		95.74 ±0.27	51.78 ±0.47	21.82 ±0.49	70.75 ±1.80	32.84 ±0.62	1.00[1] ±0.20	90.54 ±1.78	42.91 ±1.16	21.42 ±0.49
√	√	√	**96.55** ±0.16	52.55 ±0.69	24.41 ±0.57	**96.84** ±0.18	**57.82** ±0.61	24.22[2] ±0.11	89.28 ±1.61	**56.74** ±1.31	28.17 ±0.47

Notes
[1] Very low stability with gradient explosion without further preventive measures
[2] High gains in stability due to weight splitting

and with our biological modifications applied through the BioNet Converter. Tables 6 and 7 show the results presented in the main papers' Figures 7(a) and (b).

Fuzzy Learning Rates Sampling Distributions

Here, we present out observations we made on applying different distributions to sample the learning rate coefficient τ.

Experimentation

In our experiments, we evaluated the impact of fuzzy gradient scaling on neural network training using both MLP and CNN architectures on the MNIST dataset. The dataset was split into 70% training, 15% validation, and 15% test sets, with 3-fold cross-validation performed on the training data. We investigated five different probability distributions for the fuzzy gradient scaling parameter τ: uniform, normal, log-normal, geometric, and beta distributions. For each distribution, we tested 10 logarithmically spaced values of τ centered around 0.1 (ranging from 0.003 to 1.0). Both network architectures maintained consistent baseline structures, with the MLP using layers of 128 hidden neurons

Supplementary Table 4: Comparison of Models with and without Bio Modifications analyzed with torch.profiler. The table shows different metrics including parameters, FLOPs, CPU Memory usage in MB, and CPU processing time in seconds for both biomod and standard versions of different neural network architectures.

Model	Parameters Count		FLOPs Operations		CPU Mem (MB) Usage		CPU Time (s) Duration	
	Bio	Not Bio	Bio	Not Bio	Bio	Not Bio	Bio	Not Bio
AlexNet	3,786,468	3,786,468	191,471,616	191,471,616	61,509,472	63,775,616	37,281	37,281
	3,786,468	3,786,468	382,943,232	382,943,232	63,775,616	63,775,616	37,281	41,070
	3,786,468	3,786,468	765,886,464	765,886,464	68,307,904	68,307,904	48,974	53,741
	3,786,468	3,786,468	1,531,772,928	1,531,772,928	77,372,480	77,372,480	45,938	45,938
	3,786,468	3,786,468	3,063,545,856	3,063,545,856	95,501,632	95,501,632	52,647	52,647
MLP	3,173,100	3,173,100	38,464,000	38,464,000	38,128,032	38,128,032	4,948	6,019
	3,173,100	3,173,100	76,928,000	76,928,000	38,183,232	38,183,232	6,019	6,019
	3,173,100	3,173,100	153,856,000	153,856,000	38,493,840	38,493,840	6,591	6,591
	3,173,100	3,173,100	307,712,000	307,712,000	38,514,432	38,514,432	9,587	9,587
ResNet	65,010,640	65,010,640	76,620,508,160	76,620,508,160	2,142,148,592	2,142,148,592	583,724	5,919
	65,010,640	65,010,640	153,241,016,320	153,241,016,320	3,260,888,512	3,260,888,512	994,524	6,019
	65,010,640	65,010,640	306,482,032,640	306,482,032,640	5,468,873,712	5,468,873,712	1,729,510	1,729,510
	65,010,640	65,010,640	612,964,065,280	612,964,065,280	14,290,782,208	14,290,782,208	3,328,090	3,328,090

Supplementary Table 5: Results of the experiment on gradient inversion. The table shows the observed reconstruction error (mean square error) for different combinations of methods for untrained models and models trained for 100 epochs on CIFAR-100. Highest reconstruction errors for the dataset and architecture combinations are highlighted in bold. The numbers FL, WR and WS denote the methods of fuzzy learning rates, weight rejuvenation, and weight splitting respectively.

Methods			MLP MSE[%]↑		AlexNet MSE[%]↑		ResNet20 MSE[%]↑		ResNet32 MSE[%]↑	
**FL**	**WR**	**WS**	0 ep	100 ep	0 ep	100 ep	0 ep	100 ep	0 ep	100 ep
			3.47	3.62	0.22	0.20	62.20	73.65	70.78	79.61
		√	30.21	31.26	28.30	30.33	88.76	89.19	94.31	94.71
	√		3.06	4.31	0.26	0.21	59.07	62.21	70.58	81.14
	√	√	41.42	45.04	28.38	39.06	75.93	85.49	81.34	84.37
√			**55.53**	60.05	42.11	46.96	49.93	50.05	75.47	78.71
√		√	42.70	58.73	94.47	96.85	81.68	86.74	97.31	102.43
√	√		47.74	55.30	35.43	53.60	59.07	62.21	94.52	107.36
√	√	√	49.65	**64.84**	**101.79**	**112.85**	**124.21**	**132.17**	**135.35**	**155.43**

and the CNN employing two convolutional layers (32 and 64 filters) followed by two fully connected layers. The training was conducted using the Adam optimizer with a learning rate of 0.001 for 50 epochs per configuration. Results indicate that the optimal τ values varied significantly across distributions, with log-normal and beta distributions generally showing more stable performance across different scaling factors. The CNN architecture demonstrated greater robustness to variations in τ compared to the MLP, particularly for larger scaling parameter values.

Results

Our experiments show that fuzzy gradient scaling is a certain way to improve performance for both architectures (cp. Figs. 2 and 3). The baseline MLP achieved 98.6% accuracy on MNIST, while a CNN reached 99.4%. All tested

Supplementary Table 6: Results on tuned benchmark settings for the models ResNeXt28, WResNet28x10, EfficientNet V2M in combination with the datasets CIFAR10, CIFAR100 and Tiny ImageNet.

Model	CIFAR10 acc [%]↑	CIFAR100 acc [%]↑	Imagewoof acc [%]↑	Tiny ImageN. acc [%]↑
ResNeXt(29/50) 32x4d[1]	96.75	84.06	87.96	57.39
WResNet(28/50)x10[1]	97.18	84.45	89.15	55.89
EfficientNet V2 M	89.22	54.41	79.54[3]	30.86
BioResNeXt(29/50) 32x4d[2]	96.81	84.36	89.00	58.01
BioWResNet(28/50)x10[2]	97.20	84.65	89.79	56.13
BioEfficientNet V2 M[2]	89.92	61.61	83.18[3]	36.92
SeResNeXt[2]	92.05	68.44	92.16	57.14
BioSeResNeXt [2]	92.54	69.58	92.42	59.12
SwinTrans V2 t [2]	89.83	73.2	97.1	77.4
BioSwinTrans V2 t [2]	90.42	74.1	97.5	79.1

Notes
[1] Input size dependent network depth: 28 (CIFAR), 50 (ImageWoof, TinyImageNet)
[2] PyTorch implementation of the model converted by BioNet Converter
[3] Reduced batchsize (bs=65) due to lack of GPU Memory (80 GB)

distributions showed statistically significant improvements over the no-fuzziness baseline, with markedly stronger effects in the CNN architecture. Cohen's d is the standardized difference between two means, calculated by the mean difference divided by the pooled standard deviation. The typical interpretations are: d = 0.2, 0.5, and 0.8 correspond to small, medium, and large effects, respectively. Tests of statistical significance were conducted using two-sample t-tests at $p < 0.01$ between each fuzzy configuration and the baseline. The uniform distribution thus showed the strongest improvement, with large effect sizes (d = up to 1.9 for CNN, d = up to 0.84 for MLP) across several scaling rates . Correspondingly, these substantial effect sizes represent differences of almost two standard deviations between fuzzy and non-fuzzy training within some configurations - an enormous practical effect. The ideal scaling rate would seem to lie around $\tau = 0.077$, from which we reach peak performance across several distributions. While all distributions presented benefits, the uniform distribution is particularly appealing due to its consistent performance and strong effect sizes. The lognormal distribution also looked promising, especially in the CNN architecture (d up to 1.27), but with more variability across scaling rates. Beta and geometric distributions showed moderate but reliable improvements (d typically between 0.6 and 1.0), suggesting they are viable alternatives. We recommend the uniform distribution to be the default choice for future experiments and perform a hyper-parameter search based on this observations, given its strong and consistent performance in both

Supplementary Table 7: Time-series prediction results for the chaotic, high dimensional regression tasks Lorenz'96 and Thomas attractor for FDN, LSTM and GRU.

Model	Lorenz'96<br>396 dimensions<br>lyap 20 dt 0.01<br>NRMSE↓	Thomas<br>3 dimensions<br>lyap 0.76 dt 0.005<br>NRMSE↓
LSTM	0.57	0.38
GRU	0.59	0.38
Transformer	0.50	0.98
FDN[1]	0.15	0.37
BioLSTM	0.52	0.36
BioGRU	0.57	0.36
BioTransformer	0.44	0.87
BioFDN[1]	0.11	0.35

Notes

[1] Architecture introduced by Hofmann et. al [2].

architectures. The highest observed effect sizes and broadest range of significant improvements across scaling rates justify this recommendation. However, if computational resources are not an issue, the lognormal distribution might be worth exploring as an alternative, particularly for CNN architectures, as it performs competitively at several scaling rates. The consistent positive significant effect sizes across distributions and architectures serve to reinforce that fuzzy gradient scaling is indeed valuable for improving neural network training, with the choice of distribution being far less critical than the inclusion of fuzziness itself.

Dale's Principle

Dale's principle, which states that neurons release the same neurotransmitter at all their synapses [3], was a foundational concept in neuroscience but has been challenged by extensive evidence of co-transmission [4].

Recent research shows that multi-transmitter neurons are more common than previously assumed, challenging Dale's principle [4, 5]. In the supramammillary-hippocampal pathway, over 50% of terminals exhibit dual transmitter capability, enabling sophisticated frequency-dependent filtering of neural signals [6]. These neurons enhance information processing by allowing dynamic switching between excitation and inhibition based on activity patterns [7]. Evidence from human cortical studies suggests similar prevalence and functionality in higher cognitive regions [8], indicating multi-transmitter neurons are fundamental components of learning and memory circuits rather than rare exceptions. Early observations in sympathetic neurons showed nearly

Supplementary Table 8: Detailed comparison of log-normality test results, weight distribution statistics, and final validation accuracies for neural networks trained with various method combinations. FL (Fuzzy Learning), WR (Weight Rejuvenation), and WS (Weight Splitting) are evaluated both with and without Dale's Principle (DP). The table includes Shapiro–Wilk (SW) and D'Agostino's K^2 (K^2) p-values to assess log-normality, alongside skewness and kurtosis for log-transformed weights. Validation accuracies indicate the effectiveness of each method in mitigating DP-induced impairments and optimizing learning. A very low p-value denotes significant deviation from log-normality, while skew and kurtosis reflect asymmetry and tail heaviness in weight distributions. Biomod shows the highest accuracy improvements under both DP-constrained and unconstrained settings.

FL	WR	WS	DP	SW p-value	K^2 p-value	Skew	Kurtosis	Acc [%]
-	-	-	-	2.00e-06	6.4e-05	-1.29	1.14	96.18
-	-	X	-	0.02	0.03	-0.74	0.23	96.78
-	X	-	-	9.3e-08	1.9e-10	-0.32	-1.36	97.02
-	X	X	-	1.6e-08	3.9e-39	-0.42	-1.58	97.42
X	-	-	-	2.44e-08	1.8e-07	-1.70	2.41	97.68
X	-	X	-	0.01	0.04	-0.70	0.02	97.63
X	X	-	-	4.4e-08	7.2e-65	-0.27	-1.63	97.94
X	X	X	-	3.5e-08	1.7e-24	-0.44	-1.52	98.17
-	-	-	X	3.4e-09	0	0.02	-1.77	89.68
-	-	X	X	8.3e-10	8.8e-228	0.02	-1.86	92.84
-	X	-	X	1.5e-12	0.001	1.17	-0.42	87.34
-	X	X	X	5.5e-12	0.001	0.97	-0.86	91.42
X	-	-	X	2.2e-08	6.7e-231	0.01	-1.69	92.33
X	-	X	X	8.7e-10	2.4e-229	0.02	-1.85	92.48
X	X	-	X	1.5e-12	0.001	1.15	-0.50	91.58
X	X	X	X	5.5e-12	0.001	0.98	-0.85	94.32

complete co-transmission capability [9], supporting the biological plausibility of neural networks with mixed excitatory-inhibitory connections.

Formalization

For each dense layer with N output neurons, we assign $\lfloor p_E N \rfloor$ excitatory and $\lfloor p_I N \rfloor$ inhibitory neurons, where $p_E + p_I \leq 1$. The remaining neurons are mixed. A type vector $T \in \{0, 1, 2\}^N$ tracks assignments:

$$T_n = \begin{cases} 0, & \text{excitatory (E)}, \\ 1, & \text{inhibitory (I)}, \\ 2, & \text{mixed (M)}. \end{cases}$$

During initialization and after each gradient update, weights are clamped:

$$w_{n,i} \leftarrow \begin{cases} \max(0, w_{n,i}), & \text{if } T_n = 0, \\ \min(0, w_{n,i}), & \text{if } T_n = 1, \\ w_{n,i}, & \text{if } T_n = 2. \end{cases} \tag{1}$$

To compensate for reduced weight flexibility under clamping, initial weights are scaled by $\sqrt{2}$ relative to standard initialization schemes. These constraints

apply to dense layers (fully connected and convolutional), as they most closely parallel biological neural connectivity.

Code Implementation

The provided algorithm (cp. Algorithm 1) assigns each neuron to excitatory, inhibitory, or mixed based on user-defined percentages (`excit_pct` and `inhib_pct`). A small fraction (e.g., 5%) remain *mixed*, which proved critical for stable learning. The function `partial_enforce_dales_principle` then applies Equation 1 after each gradient update. This ensures that each neuron's row in $\mathbf{W}$ preserves its sign constraints throughout training.

Bias Terms

The bias term is unconstrained or slightly shifted to accommodate changes in activation distributions. In our implementation, excitatory neurons can be given a small positive bias, inhibitory neurons a small negative bias, and mixed neurons a neutral bias. Allowing the bias to vary helps offset any shift in the post-synaptic activation that arises from imposing sign constraints on the weights.

Biological and Empirical Observations

Most cortical neurons are purely excitatory or purely inhibitory [6, 8], but a small subset in some brain regions exhibits co-release or mixed dynamics [5]. When we constrained *all* neurons to be strictly excitatory or strictly inhibitory, training frequently stalled. Introducing a small portion (e.g., 5%−10%) of mixed neurons consistently improved convergence. In addition, weight splitting (WS) proved synergistic with Dale's principle, as it preserves biologically inspired connectivity patterns without entirely sacrificing the network's capacity to learn.

Complexity and Integration

Imposing Dale's principle entails a simple clamping operation after each parameter update, which has linear complexity in the number of weights. The method integrates seamlessly with our fuzzy learning rate (FL), weight rejuvenation (WR), and weight splitting (WS) plug-ins. Together, these techniques form a biologically motivated set of constraints and transformations for modern ANN architectures.

Experimentation

We use the MNIST dataset, which contains 60,000 training images and 10,000 test images, and set aside 5,000 training images for validation, leaving 55,000 for training. Each image is normalized to a mean of 0.1307 and a standard deviation of 0.3081. Our `SimpleCNN` consists of a single convolutional layer (in-channels of 1, out-channels of 8, kernel size of 3, and padding of 1), followed by a `Swish` activation, a 2×2 max pooling step, and a fully connected layer

mapping the feature map ($8 \times 14 \times 14$) to 10 output classes, with a final log-softmax for classification. Dale's principle is enforced by clamping excitatory weights to nonnegative and inhibitory weights to nonpositive after each gradient update. We train for 1,289 steps using a batch size of 128, a learning rate of 0.1, and no momentum. Each step involves a forward pass, backpropagation, an optimizer update, and a Dale's clamping operation. We track validation accuracy every 50 steps and report the final performance on a separate test set.

Results

Here, we present the additional results regarding the integration of Dales's Principle into ANNs.

Weight Distributions

Analysis of weight distributions across methodological configurations reveals systematic relationships between distributional characteristics and model performance. The baseline configuration (FL– WR– WS– DP–) demonstrates significant deviation from log-normality, as evidenced by Shapiro–Wilk (2.96e-06) and K^2 (6.4e-05) test statistics, exhibiting moderate negative skew (-1.3) and mild positive kurtosis (1.1363), as detailed in Table 8. These distributional properties correspond to suboptimal network performance, establishing a reference point for method-induced improvements. Individual method applications transform these distributions systematically. Weight splitting in isolation reduces skew magnitude (-0.7) while normalizing kurtosis (0.23), improving alignment with log-normal characteristics. The FL+WS configuration achieves near-optimal distributional properties (skew = -0.7, kurtosis = 0.02) with borderline-significant log-normality measures (Shapiro–Wilk = 0.01, K^2 = 0.1). Of particular interest, biomod attains maximum accuracy (98.17%) while maintaining moderate deviations from perfect log-normality (skew = -0.4, kurtosis = -1.5), suggesting that strict log-normality is not a prerequisite for optimal performance. Principal Component Analysis (PCA), as illustrated in Figure 4, reveals distinct clustering patterns in weight space. Configurations with multiple methods demonstrate systematic shifts in cluster centroids relative to single-method implementations, indicating fundamental alterations in synaptic organization. This structural differentiation becomes particularly pronounced with combining all three methods, suggesting synergistic effects on weight distribution characteristics. Under Dale's principle constraints, weight distributions manifest characteristically different patterns. The baseline DP configuration exhibits near-zero skew (0.02) but pronounced negative kurtosis (-1.7683), reflecting fundamental constraints on distribution symmetry. In contrast, biomod achieves optimal DP-constrained accuracy (94.32%) while maintaining moderate positive skew (0.9762) and reduced negative kurtosis (-0.8450), demonstrating the feasibility of effective learning despite distributional constraints. Neuron-level analysis, presented in Figure 5, demonstrates that FL, WR, and WS systematically increase the proportion of excitatory connections in unconstrained settings. When combined with Dale's principle, these

methods facilitate the maintenance of balanced excitatory-inhibitory distributions despite sign constraints, as evidenced by the weight fraction distributions. The QQ plots in Figure 6 provide visual confirmation of these distributional modifications, demonstrating reduced tail distortion when multiple methods are combined. These findings establish clear correlations between weight distribution characteristics and model performance, with optimal results achieved through configurations that moderate extreme tail behavior while maintaining sufficient distributional flexibility for effective learning. The systematic impact of method combinations on weight distributions, visualized through PCA projections and quantified via statistical measures, demonstrates the efficacy of combined plasticity mechanisms in shaping network architecture for enhanced performance.

Loss Landscape

In the two-dimensional contour plots Figure 7 and the corresponding three-dimensional surfaces Figure 8, each combination of Fuzzy Learning (FL), Weight Rejuvenation (WR), and Weight Splitting (WS), with or without Dale's Principle (DP), exhibits a distinct loss-surface shape characterized by different peak heights, valley depths, and topographical features. The baseline configuration without any of these methods consistently shows peaks around 6.4 in the two-dimensional color scale and slightly above 2.0 in the three-dimensional view. Its valley reaches approximately 0.2 to 0.3 in the absence of Dale's Principle and rises to about 0.4 to 0.5 when Dale's Principle is enforced. In this baseline condition, the color contours and the three-dimensional rendering both reveal a relatively compact low-loss region, and the trajectory from the initial point down into the valley follows an S-shaped path that curves around a small ridge before converging. This lateral detour suggests a saddle-like region near the top of the surface, leading to a moderately long route through parameter space even though the overall loss range (peak minus valley) is not as large as in some other configurations.

When Weight Splitting or Weight Rejuvenation is introduced individually, the maximum and minimum loss values remain close to those seen in the baseline, but the low-loss basin in each two-dimensional contour can become slightly broader, and the three-dimensional surface appears less sharply ridged. In these single-method conditions, the optimizer's path remains curved but does not exhibit as pronounced a lateral deviation as in the baseline. The resulting descent is still spread across roughly six to seven visible update steps, with the total loss drop hovering around 6.0 to 6.4 in the color scale, indicating a moderate slope overall. Fuzzy Learning alone produces a similar effect, with a peak near 6.4 and a final valley around 0.2 to 0.3 without Dale's Principle, again shifting slightly higher when Dale's Principle is applied. In all these single-method plots, the valley floor is moderately sized—bigger than the baseline in some cases—and the three-dimensional surface does not show strongly pronounced saddles, though small ridges remain in certain regions, causing gentle curvature rather than a perfectly straight descent.

Configurations that combine two methods for example, Weight Rejuvenation with Weight Splitting often present higher peak values in the two-dimensional plots (rising toward 7.0) and in the corresponding three-dimensional surfaces (reaching above 3.0 in some panels). The deepest portion of the low-loss basin can lie around 0.2 or slightly lower when Dale's Principle is not enforced, while remaining above 0.3 to 0.4 with Dale's Principle. These broader vertical ranges, combined with smoother surfaces in many of the subplots, indicate a relatively steep but more direct descent. The extent of the dark or brownish region in the two-dimensional visualizations often increases in these two-method cases, suggesting an enlarged basin that does not force the trajectory to make as many detours. In three-dimensional views, the final valleys appear flatter and wider, meaning there is more space along the bottom of the loss surface before contour lines rise again. The path traced by the optimizer in these conditions often starts with a noticeable drop from a higher plateau, transitions around or over small ridges, and settles into a valley that has gentler boundaries on either side. The approximate parameter-space distance is thus potentially large, given the higher starting peak, but the path itself is less tortuous and can appear shorter when measured by lateral deviation.

When all three methods—Fuzzy Learning, Weight Rejuvenation, and Weight Splitting—are active simultaneously, the contour plots show some of the highest peak values, occasionally above 7.5 or 8.0, paired with notably low minima near 0.15 to 0.2 without Dale's Principle. This creates the largest overall drop in loss when viewed from peak to valley. The three-dimensional surfaces in these cases tend to have broad, flatter basins, evidenced by a relatively extensive dark region near the bottom that contrasts with steep walls rising toward the higher-loss plateau. Although the total vertical distance in the color scale is large, the pathway often follows a more direct arc rather than a winding route around multiple ridges. The effective parameter-space distance can therefore be substantial due to a high starting peak and a very low final valley, but the visualization suggests that saddles or narrow ridges are less prominent, resulting in fewer lateral diversions and a smoother descent. The low-loss basin occupies a visibly larger area in the two-dimensional plots, indicating broader valleys that can accommodate a range of parameter perturbations with only a minor rise in loss. In the presence of Dale's Principle, the minimum sits somewhat higher, but the widened basin remains evident, and the characteristic broad, flattened shape of the valley is still noticeable in both the contour lines and the three-dimensional surface.

Catastrophic Forgetting

Experimentation

We trained four distinct methodologies— CBP (continuous backprob), L2 (l2 norm regularization), biomod (FL+WR+WS), and biomod + continuous backprob, CBP—utilizing the implementation provided by [10] (cp. https:

//github.com/shibhansh/loss-of-plasticity) on ImageNet-style tasks encompassing up to 1000 classes in total, with few classes per task trained sequentially. The experimental parameters, including epoch count, mini-batch size, network architectures, and associated hyperparameters (weight decay, momentum, and related parameters), are transparently documented in the provided codebase. Our standard configuration employed 5000 epochs, utilized a mini-batch size of 100, and implemented either a standard SGD optimizer or its close variants.

Given the computational intensity of these experiments, we conducted minimal hyperparameter optimization, as a single experimental run demanded no less than 36 hours on an NVIDIA A40 GPU at full utilization. With 30 repetitions per method across four distinct methodologies, our total GPU computation amounted to

$$36\,\text{h/run} \times 30\,\text{runs} \times 4\,\text{methods} \;=\; 4320\,\text{hours},$$

translating to approximately 180 days of continuous GPU computation. For this computational setup, we additionally considered CPU overhead and estimated the total power consumption at approximately 400 W. This culminated in an aggregate energy consumption of roughly 1728 kWh, corresponding to a carbon footprint of approximately 0.86 tonnes CO_2, contingent upon regional power grid emission factors.

The core training procedure, implemented within the `repeat_expr` function, encompasses several key components. The procedure begins with data shuffling at each epoch, followed by mini-batch feeding to the designated learning method. Subsequently, both training and test accuracies are computed via the `nll_accuracy` metric. Network architectures (e.g., `ConvNet` versus `ConvNet2`) are initialized according to their specifications, with the `BioConverter` being applied specifically for biomod variants. The `BioConverter` itself is parameterized with several crucial parameters. The fuzzy learning rate factor nu is set to 0.13, while the dampening factor is configured at 0.3. The rejuvenation parameter dre is set to 14.0. The weight splitting parameter Gamma is defined as 2, and Dale's principle application is set to False. The base learning rate is initialized at 0.01, the stability factor is configured at 3.0, and the learning rate variability is set to 0.1.

Results

Our experimental analysis demonstrates distinct performance characteristics across the four methodologies—CBP, L2, biomod, and biomod+CBP—over 5×10^3 training iterations. The learning trajectories can be characterized into three phases: an initial rapid improvement, an intermediate refinement, and a final convergence. Quantitatively, all methods exhibited comparable starting performance around 84% accuracy during the initial phase (0–200 iterations). biomod and biomod+CBP displayed accelerated early learning, achieving 87–88% accuracy, while CBP and L2 reached 86–87%. This divergence in early performance patterns became statistically significant ($p < 0.05$) by iteration 200.

The intermediate range (200–2000 iterations) presented a consistent, hierarchical pattern among performances: biomod maintained superior performance, fluctuating between 88–90%, while biomod+CBP closely followed at 88–89%. CBP performed marginally lower but remained competitive (87–89%), while L2 regularization consistently underperformed, maintaining 87–88.5% accuracy. The performance differential between biomod and L2 remained statistically significant throughout this phase ($p < 0.01$). Most noticeably, during the peak performance phase (2000–3000 iterations), we observed remarkable spikes in accuracy. Both biomod and biomod+CBP frequently exceeded 90% accuracy, with biomod+CBP achieving a maximum of 90.8% at approximately iteration 2350. Variability in performance, represented by confidence intervals, exhibited method-specific characteristics: L2 and biomod showed wider variance bands ($\pm 1.2\%$ and $\pm 1.1\%$ respectively), whereas CBP and biomod+CBP demonstrated more stable performance ($\pm 0.8\%$ and $\pm 0.9\%$, correspondingly).

In the convergence phase (3000–5000 iterations), all methods reached stability within a narrow performance band: Biomod, biomod+CBP, and CBP converged to approximately 90–91% accuracy, while L2 stabilized at 89%.The computational demands of our experimentation were substantial, requiring 4.32×10^3 GPU hours (180 days) of continuous computation on NVIDIA A40 hardware. This resulted in an estimated energy consumption of 1.728×10^3 kWh and a carbon footprint of approximately 0.86 tonnes CO_2.

Used Metrics

This section provides detailed definitions of the metrics used in our analysis.

Error Rate

The error rate (E) is calculated as the proportion of incorrect predictions to the total number of predictions:

$$E = \frac{\text{Number of incorrect predictions}}{\text{Total number of predictions}} = \frac{FP + FN}{TP + TN + FP + FN} \tag{2}$$

where FP represents false positives, FN false negatives, TP true positives, and TN true negatives.

Accuracy

The accuracy (A) measures the proportion of correct predictions:

$$A = \frac{\text{Number of correct predictions}}{\text{Total number of predictions}} = \frac{TP + TN}{TP + TN + FP + FN} \tag{3}$$

Normalized Area Under Curve

The Normalized Area Under Curve (nAUC) provides a measure of learning speed and stability. It is calculated as the mean accuracy across all training epochs:

$$\text{nAUC} = \frac{1}{N}\sum_{i=1}^{N} A_i \tag{4}$$

where N is the total number of epochs and A_i is the accuracy at epoch i.

Mean Square Error

The Mean Square Error (MSE) measures the average squared difference between predicted ($\hat{y}_i$) and actual (y_i) values:

$$\text{MSE} = \frac{1}{n}\sum_{i=1}^{n}(y_i - \hat{y}_i)^2 \tag{5}$$

where n is the number of samples.

Normalized Root Mean Square Error

The Normalized Root Mean Square Error (NRMSE) is the root mean square error normalized by the range of observed values:

$$\text{NRMSE} = \frac{\sqrt{\frac{1}{n}\sum_{i=1}^{n}(y_i - \hat{y}_i)^2}}{y_{\max} - y_{\min}} \tag{6}$$

where $y_{\max}$ and $y_{\min}$ are the maximum and minimum observed values respectively.

Statistical Moments

To analyze the distribution of our results, we calculated the following statistical moments:

Skewness

Skewness (γ_1) measures the asymmetry of the probability distribution:

$$\gamma_1 = \frac{\mathbb{E}[(X-\mu)^3]}{\sigma^3} = \frac{\frac{1}{n}\sum_{i=1}^{n}(x_i - \bar{x})^3}{(\frac{1}{n}\sum_{i=1}^{n}(x_i - \bar{x})^2)^{3/2}} \tag{7}$$

where μ is the mean, σ is the standard deviation, and $\bar{x}$ is the sample mean.

Kurtosis

Kurtosis (γ_2) measures the "tailedness" of the probability distribution:

$$\gamma_2 = \frac{\mathbb{E}[(X-\mu)^4]}{\sigma^4} = \frac{\frac{1}{n}\sum_{i=1}^{n}(x_i - \bar{x})^4}{(\frac{1}{n}\sum_{i=1}^{n}(x_i - \bar{x})^2)^2} \tag{8}$$

Cohen's d Effect Size

Cohen's d (d) is a standardized measure of the difference between two group means, calculated as:

$$d = \frac{\bar{x}_1 - \bar{x}2}{s_{\text{pooled}}} \tag{9}$$

where $\bar{x}1$ and $\bar{x}_2$ are the means of the two groups, and s_{pooled} is the pooled standard deviation:

$$s_{\text{pooled}} = \sqrt{\frac{(n_1 - 1)s_1^2 + (n_2 - 1)s_2^2}{n_1 + n_2 - 2}} \tag{10}$$

where n_1 and n_2 are the sample sizes, and s_1^2 and s_2^2 are the variances of the two groups.

Two-Sample t-Test

The two-sample t-test evaluates whether the means of two independent groups are significantly different. The test statistic (t) is calculated as:

$$t = \frac{\bar{x}_1 - \bar{x}_2}{\sqrt{\frac{s_1^2}{n_1} + \frac{s_2^2}{n_2}}} \tag{11}$$

where $\bar{x}_1$ and $\bar{x}_2$ are the sample means, s_1^2 and s_2^2 are the sample variances, and n_1 and n_2 are the sample sizes of the two groups. The degrees of freedom (df) for this test can be approximated using the Welch–Satterthwaite equation:

$$df = \frac{(\frac{s_1^2}{n_1} + \frac{s_2^2}{n_2})^2}{\frac{(s_1^2/n_1)^2}{n_1 - 1} + \frac{(s_2^2/n_2)^2}{n_2 - 1}} \tag{12}$$

References

[1] Rapin, J. & Teytaud, O. Nevergrad - A gradient-free optimization platform. https://GitHub.com/FacebookResearch/Nevergrad (2018).

[2] Hofmann, M. & Mäder, P. Synaptic scaling–an artificial neural network regularization inspired by nature. *IEEE Transactions on Neural Networks and Learning Systems* 1–15 (2021). https://doi.org/10.1109/TNNLS.2021.3050422 .

[3] Dale, H. Pharmacology and nerve-endings. *Proceedings of the Royal Society of Medicine* **28** (3), 319–332 (1935). https://doi.org/10.1177/003591573502800330 .

[4] Granger, A. J., Wallace, M. L. & Sabatini, B. L. Multi-transmitter neurons in the nervous system. *CURRENT OPINION IN NEUROBIOLOGY* **45**, 85–91 (2017). https://doi.org/10.1016/j.conb.2017.04.007 .

[5] Vaaga, C. E., Borisovska, M. & Westbrook, G. L. Dual-transmitter neurons: functional implications of co-release and co-transmission. *CURRENT OPINION IN NEUROBIOLOGY* **29**, 25–32 (2014). https://doi.org/10.1016/j.conb.2014.04.010 .

[6] Hirai, H. *et al.* Distinct release properties of glutamate/gaba co-transmission serve as a frequency-dependent filtering of supramammillary inputs. *ELIFE* **13** (2024). https://doi.org/10.7554/eLife.99711 .

[7] Borisovska, M., Bensen, A. L., Chong, G. & Westbrook, G. L. Distinct modes of dopamine and gaba release in a dual transmitter neuron. *JOURNAL OF NEUROSCIENCE* **33** (5), 1790–1796A (2013). https://doi.org/10.1523/JNEUROSCI.4342-12.2013 .

[8] Zilles, K. & Palomero-Gallagher, N. Multiple transmitter receptors in regions and layers of the human cerebral cortex. *FRONTIERS IN NEUROANATOMY* **11** (2017). https://doi.org/10.3389/fnana.2017.00078 .

[9] POTTER, D., LANDIS, S., MATSUMOTO, S. & FURSHPAN, E. Synaptic functions in rat sympathetic neurons in microcultures .2. adrenergic cholinergic dual status and plasticity. *JOURNAL OF NEUROSCIENCE* **6** (4), 1080–1098 (1986) .

[10] Dohare, S. *et al.* Loss of plasticity in deep continual learning. *Nature* **632**, 768—774 (2024) .

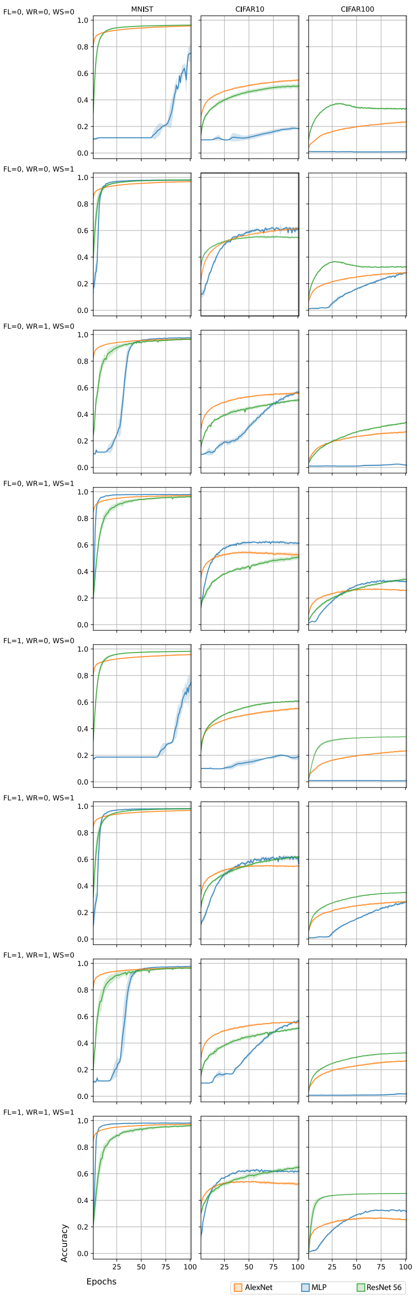

Supplementary Fig. 1: Training accuracy curves comparing the learning dynamics of AlexNet (orange), MLP (blue), and ResNet56 (green) architectures across MNIST, CIFAR10, and CIFAR100 datasets under different combinations of biological modifications (FL: Fuzzy Learning, WR: Weight Rejuvenation, WS: Weight Splitting). Each row represents a different combination of modifications, showing the evolution of accuracy over 100 epochs. Orange lines represent AlexNet, blue lines represent MLP, and green lines represent ResNet56 performance.

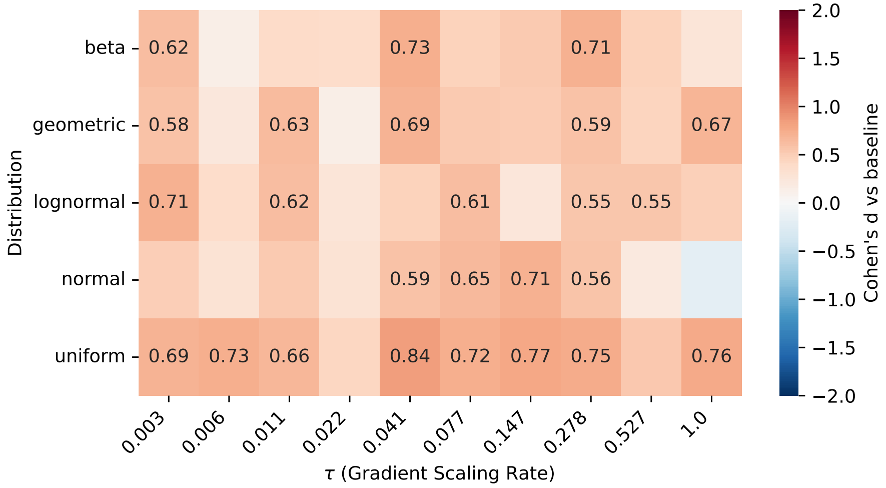

Supplementary Fig. 2: Effect sizes (Cohen's d) for different τ fuzzy gradient scaling distributions compared to baseline (no gradient scaling) on MLP architecture trained on MNIST. Values shown only where statistically significant ($p < 0.01$). Positive values (red) indicate superior performance compared to baseline.

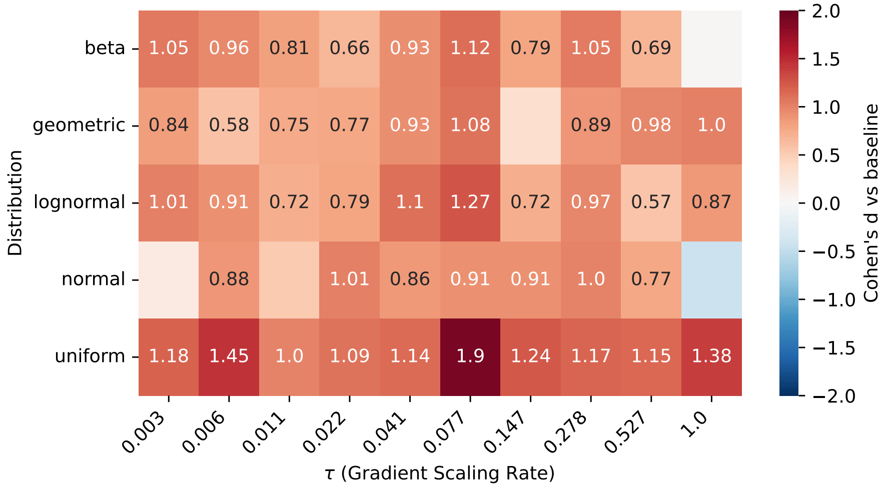

Supplementary Fig. 3: Effect sizes (Cohen's d) for different τ fuzzy gradient scaling distributions compared to baseline (no gradient scaling) on CNN architecture trained on MNIST. Values shown only where statistically significant ($p < 0.01$). Positive values (red) indicate superior performance compared to baseline.

Algorithm 1: Dale's Principle Implementation for Neural Networks

Input : Network $\mathcal{N}$, fractions $p_E, p_I \in [0, 1]$ for excitatory/inhibitory neurons
Assert : $p_E + p_I \leq 1$
Output : Network with Dale's principle enforced

```
Function InitializeDale($\mathcal{N}, p_E, p_I$):
    for each layer $L \in \mathcal{N}$ do
        if isDenseLayer(L) then
            $N_{\text{out}} \leftarrow \text{outputDim}(L)$; $n_E \leftarrow \lfloor p_E N_{\text{out}} \rfloor$; $n_I \leftarrow \lfloor p_I N_{\text{out}} \rfloor$
            $n_M \leftarrow N_{\text{out}} - n_E - n_I$
            $T \leftarrow \text{concatenate}([0]^{n_E}, [1]^{n_I}, [2]^{n_M})$; $T \leftarrow \text{shuffle}(T)$
            $L$.neuronTypes $\leftarrow T$
            $W \leftarrow \text{KaimingInit}(L) \times \sqrt{2}$
            for $i \leftarrow 0$ to $N_{out} - 1$ do
                if $T[i] = 0$ then
                    $W[i, :] \leftarrow \max(0, W[i, :])$
                else if $T[i] = 1$ then
                    $W[i, :] \leftarrow \min(0, W[i, :])$
            $L$.weights $\leftarrow W$
            if L has bias then
                $b \leftarrow \text{zeros}(N_{\text{out}})$
                for $i \leftarrow 0$ to $N_{out} - 1$ do
                    if $T[i] = 0$ then
                        $b[i] \leftarrow 0.1$
                    else if $T[i] = 1$ then
                        $b[i] \leftarrow -0.1$
                $L$.bias $\leftarrow b$

Function EnforceDale($\mathcal{N}$):
    for each layer $L \in \mathcal{N}$ do
        if isDenseLayer(L) and hasattr(L, 'neuronTypes') then
            $W \leftarrow L$.weights; $T \leftarrow L$.neuronTypes
            for $i \leftarrow 0$ to $len(T) - 1$ do
                if $T[i] = 0$ then
                    $W[i, :] \leftarrow \max(0, W[i, :])$
                else if $T[i] = 1$ then
                    $W[i, :] \leftarrow \min(0, W[i, :])$
            $L$.weights $\leftarrow W$

while not converged do
    Forward pass through $\mathcal{N}$
    Compute gradients via backpropagation
    Update weights using optimizer
    EnforceDale($\mathcal{N}$)
```

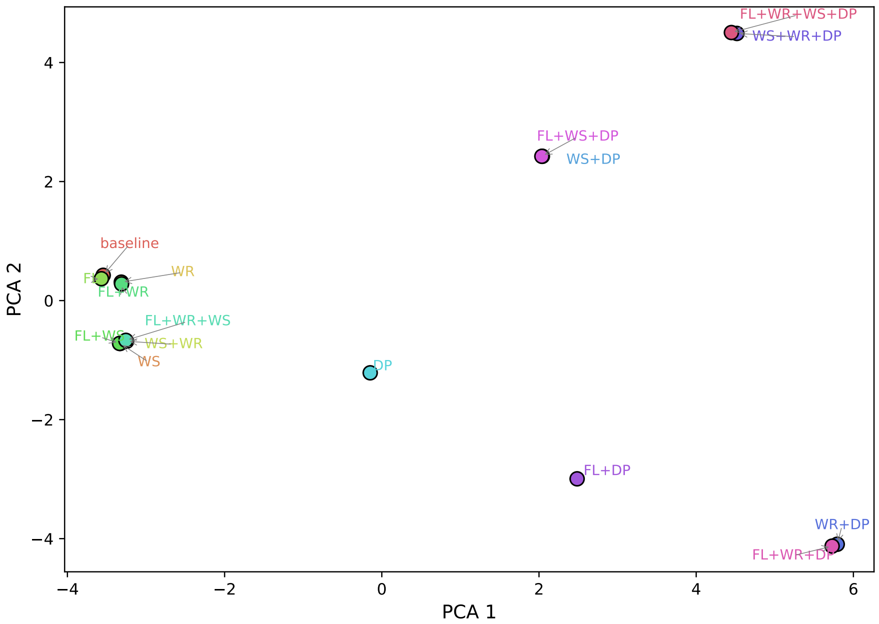

Supplementary Fig. 4: PCA visualization revealing distinct weight distribution patterns across different training methodologies (FL, WR, WS, DP and their combinations). The two-dimensional projection demonstrates clear clustering of models based on their synaptic organization, with particularly pronounced separation when Weight Rejuvenation (WR) and Fuzzy Learning (FL) are combined. This visualization highlights how biological modifications fundamentally alter the network's internal representation structure. Colors denote different combinations.

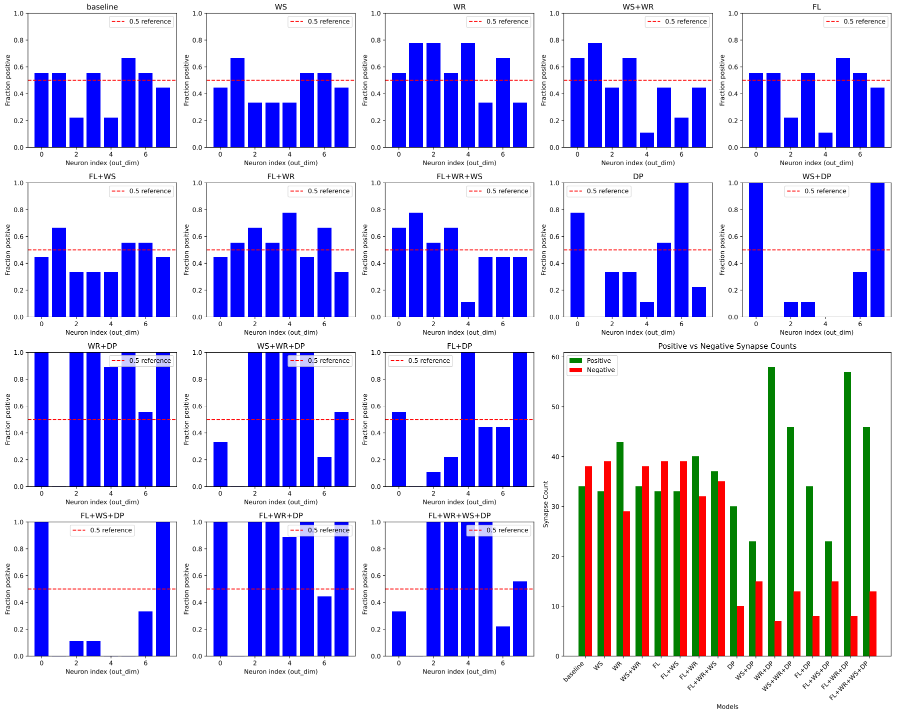

Supplementary Fig. 5: Dale's Principle adherence and synaptic balance in neural networks trained with different methods. The fraction of positive weights per neuron (top) and positive-negative synapse counts (bottom) depict adherence to Dale's Principle. The inclusion of methods like FL and WR mitigates DP's impact, maintaining functional balance and learning capacity. Green denotes positive and red negative values.

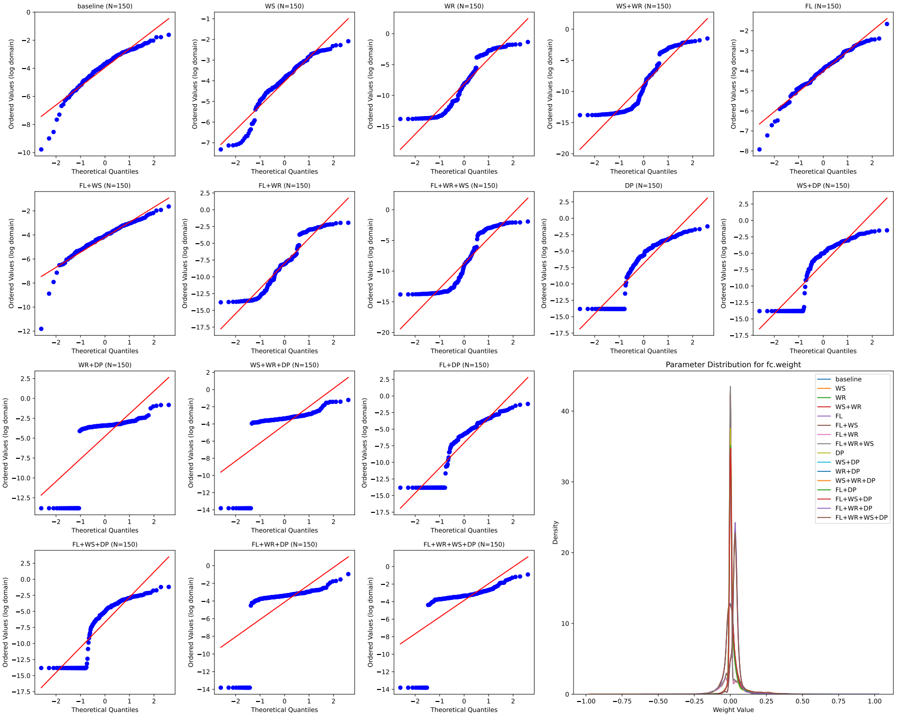

Supplementary Fig. 6: QQ plots of log-transformed weight distributions for neural networks trained with various method combinations. The plots compare empirical weight distributions against theoretical quantiles under log-normal assumptions. Models combining FL, WR, and WS demonstrate improved adherence to log-normality while balancing deviations that favor learning. Colors denote different combinations.

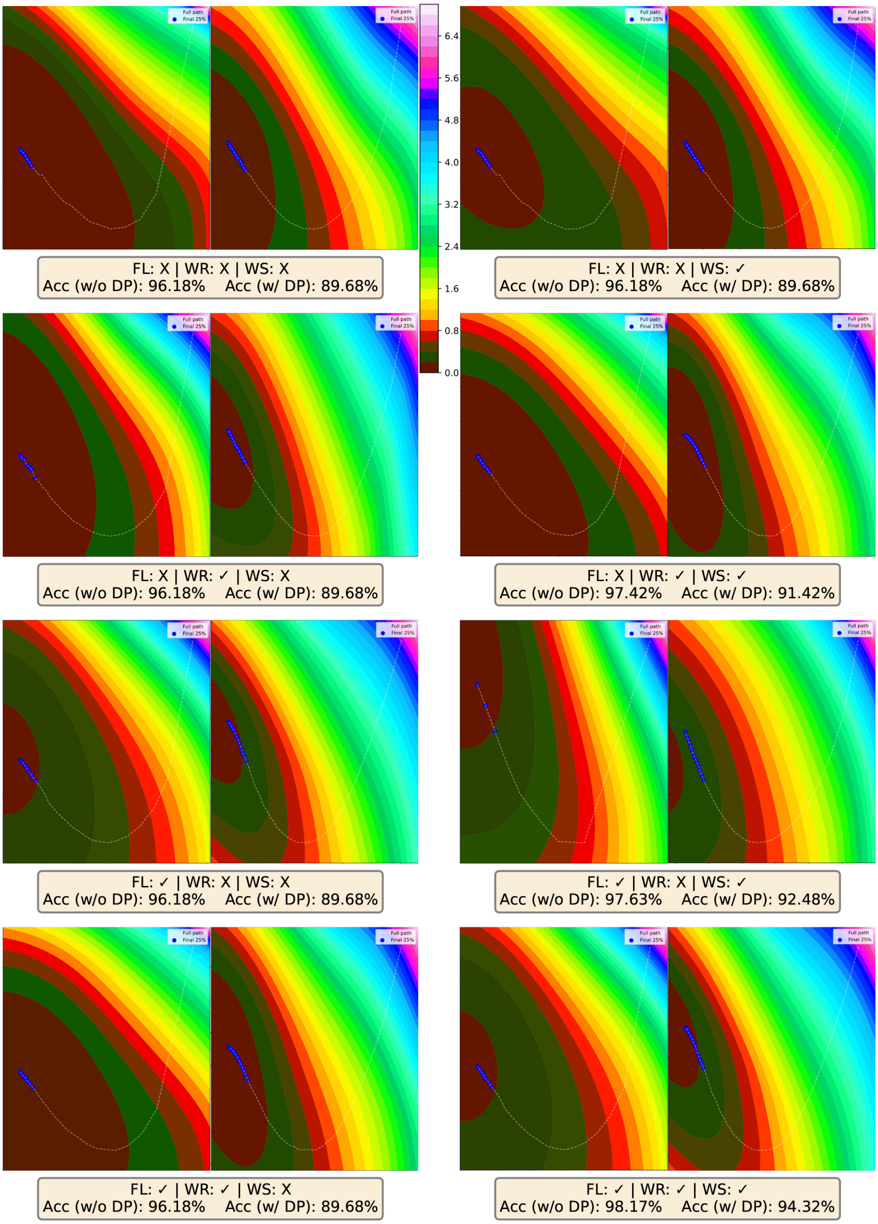

Supplementary Fig. 7: Comparative analysis of neural network optimization trajectories under various method combinations. Each subplot represents a unique configuration of Fuzzy Learning (FL), Weight Rejuvenation (WR), and Weight Splitting (WS), with and without Dale's Principle (DP). Accuracy values demonstrate method effectiveness in mitigating performance degradation introduced by Dale's Principle.

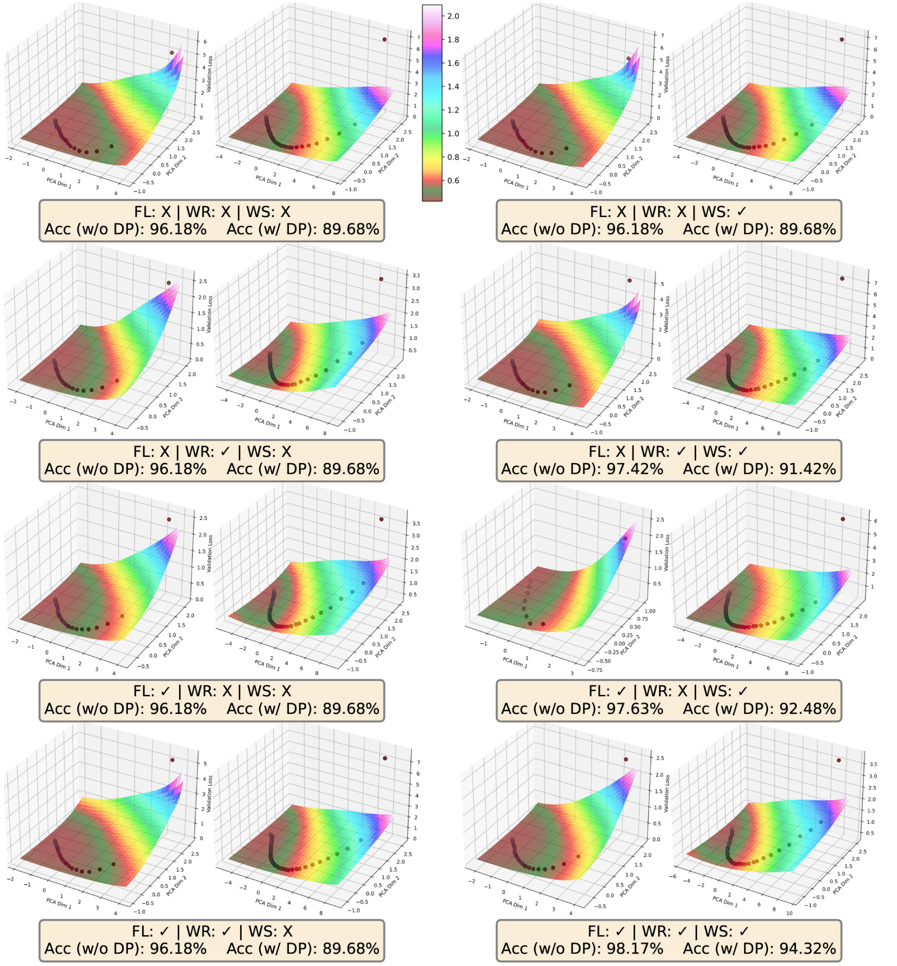

Supplementary Fig. 8: Three-dimensional visualization of neural network loss landscape across different method combinations. Subplots illustrate the impact of Fuzzy Learning (FL), Weight Rejuvenation (WR), and Weight Splitting (WS) on network optimization trajectories, comparing performance with and without Dale's Principle (DP). Accuracy percentages reveal method-specific improvements in network training.

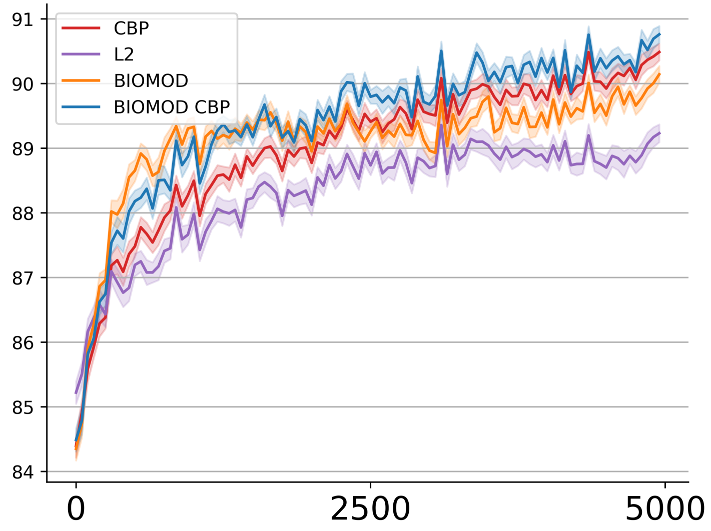

Supplementary Fig. 9: Comparison of learning trajectories for four training methodologies—Continuous Backpropagation (CBP in red), L2 regularization (L2, in purple), biomod (combination of Fuzzy Learning [FL], Weight Rejuvenation [WR], and Weight Splitting [WS] in orange), and biomod combined with CBP (biomod+CBP in blue). The graph illustrates accuracy progression over 5000 iterations, with biomod and biomod+CBP showing accelerated early learning and superior peak performance, reaching over 90% accuracy. Confidence intervals highlight method-specific stability, with biomod+CBP exhibiting reduced variability compared to L2 and CBP. The results demonstrate biomod+CBP's enhanced learning capacity while maintaining robustness across training phases. Light colored regions denote deviations.
